# Licorice processing involving functions of Evodiae Fructus on liver inflammation and oxidative stress are associated with intestinal mucosal microbiota

**DOI:** 10.3389/fmicb.2024.1439204

**Published:** 2024-08-08

**Authors:** Xuejuan Liang, Qixue Tian, Linglong Chen, Yanbing Zhang, Yanmei Peng

**Affiliations:** ^1^Hunan Academy of Chinese Medicine, Changsha, China; ^2^Hunan Provincial Hospital of Integrated Traditional Chinese and Western Medicine, Changsha, China; ^3^National Traditional Chinese Medicine Processing Technology Inheritance Base of the Affiliated Hospital of Hunan Academy of Traditional Chinese Medicine, Changsha, China; ^4^Cili County Chinese Medicine Hospital, Zhangjiajie, China

**Keywords:** small-flowered Evodiae Fructus, medium-flowered Evodiae Fructus, specification, licorice processing, liver inflammation, oxidative stress, intestinal mucosal microbiota

## Abstract

**Background:**

This study aimed to investigate the effects of licorice processing of different Evodiae Fructus (EF) specifications on liver inflammation and oxidative stress associated with the intestinal mucosal microbiota.

**Materials and methods:**

The 25 Kunming mice were divided into control (MCN), raw small-flowered Evodiae Fructus (MRSEF), raw medium-flowered EF (MRMEF), licorice-processed small-flowered EF (MLSEF), and licorice-processed medium-flowered EF (MLSEF) groups. The EF intervention groups were given different specifications of EF extract solutions by gavage. After 21 days, indices of liver inflammation and oxidative stress and intestinal mucosal microbiota were measured in mice.

**Results:**

Compared with the MCN, malondialdehyde (MDA), tumor necrosis factor-α (TNF-α), and interleukin-6 (IL-6) levels were significantly increased in the MRMEF. Although the trends of oxidative stress and inflammatory indexes in the MLSEF and MLMEF were consistent with those in the raw EF groups, the changes were smaller than those in the raw EF groups. Compared to the raw EF groups, the MLSEF and MLMEF showed closer approximations of metabolic function to the MCN. The abundance of *Corynebacterium* in MRMEF was significantly lower than that in the MCN, and it was not significantly different from the MCN after licorice processing. The probiotic *Candidatus Arthromitus* was enriched in the MLSEF. The probiotic *Lactobacillus* was enriched in the MLMEF. Correlation analysis revealed significant negative correlations between IL-1β, some metabolic functions and Corynebacterium.

**Conclusion:**

The effects of medium-flowered EF on oxidative stress and inflammatory factors in the liver of mice were stronger than those of small-flowered EF. The licorice processing can reduce this difference by modulating the abundance of *Corynebacterium* and intestinal mucosal metabolic function.

## Background

Euodiae Fructus (EF) is a medicinal herb with significant therapeutic value. It is derived from the dried nearly ripe fruits of the plants *Euodia rutaecarpa* (Juss.) Benth., *Euodia rutaecarpa* (Juss.) Benth. var. *officinalis* (Dode) Huang, or *Euodia rutaecarpa* (Juss.) Benth. var. *bodinieri* (Dode) Huang (National Pharmacopoeia Committee, 2020). Natural herbal medicines exhibit characteristics such as multiple targets, multiple actions, and multiple levels of effects. In traditional Chinese medicine (TCM), EF is often used for treating gastrointestinal disorders, headaches, oral ulcers, and vomiting (Xiao et al., 2023). Modern pharmacological studies have demonstrated that EF is rich in alkaloids, lignans, and flavonoids, which possess pharmacological properties such as analgesic, anti-tumor, and anti-inflammatory effects (Xia et al., 2023).

Ever since the book “Supplementary Records of Famous Physicians,”classic Chinese medical books such as “Bencaojing Jizhu” have documented that EF has trace amounts of toxicity. Fortunately, throughout its history of medicinal use, it has been discovered that the addition of licorice juice during the processing of EF can inhibit the generation of toxic metabolites, thereby reducing its hepatotoxicity (Ren et al., 2023). For example, Zhang et al. (2018) intervened in mice with 1, 5, and 10 times clinical doses of EF and found that licorice processing significantly reduced the degree of liver pathological damage and the elevated levels of serum alanine aminotransferase (ALT), aspartate aminotransferase (AST), lactate dehydrogenase, and alkaline phosphatase. This is supported by the findings of Shan et al. (2021) that an overdose of EF altered the morphology of mouse liver, while licorice processing effectively reduced the acute toxicity of aqueous extracts, volatile oils and ethanolic extracts of EF. The liver toxicity of EF might be related to cytochrome P450 (CYP450) (Zhang et al., 2015; Liu et al., 2020; Zhang W. et al., 2021), oxidative stress (Cai et al., 2014), inflammatory response (Liao et al., 2014) and other biochemical reactions. The research on these mechanisms of the preparation of licorice processing EF is insufficient. It is necessary to explore the different functions of EF on liver inflammation and oxidative stress before and after licorice processing, that may help to explain the mechanism of processing attenuated toxin.

In recent years, it has become increasingly clear that the vast microbial community residing in the gastrointestinal tract plays a critical and intricate role in drug absorption and overall liver health (Gratz et al., 2010; Pabst et al., 2023). The intestine is an important site for the metabolism of orally administered drugs in the body, and upon entering the intestine, the drug components are first exposed to the microbiota. Therefore, the intestinal microbiota influences the transformation and absorption of certain drug components even prior to the liver first-pass effect (Džidić-Krivić et al., 2023). The intestinal microbiota play important roles in the dual effects of effectiveness and hepatotoxicity of Chinese medicine. For example, the reactive metabolite genipin dialdehyde intermediate, generated through the gut microbiota-mediated transformation of geniposide, plays a critical role in geniposide-induced liver damage (Li et al., 2019). Another example is the anticancer drug irinotecan, which, after metabolism by bacterial β-glucuronidase, can cause severe diarrhea (Kodawara et al., 2016). In fact, an animal study has shown that the prevention and treatment of colorectal cancer by EF are associated with the modulation of gut microbial metabolites and colonic epithelial signaling pathways (Wang M. X. et al., 2021). This includes promoting the enrichment of bacteria producing short-chain fatty acids and reducing the abundance of pro-inflammatory bacteria (Wang M. X. et al., 2021; Wang et al., 2023). We discuss the effects of processing EF on its efficacy and hepatotoxicity associated with the intestinal mucosal microbiota (Long et al., 2018a,b). This will lead to a better understanding of the traditional process of EF.

In traditional Chinese medicine clinics, Evodiae Fructus is divided into different specifications and grades according to the size of the fruit:small, medium, and large flowers, and the ancients hold that small-flowered EF is of superior quality. It is worth noting that the large- flowered EF is usually fully matured, and the seeds separate from the husk, which is inconsistent with the description of ‘nearly mature’ in the Chinese Pharmacopoeia (National Pharmacopoeia Committee, 2020; Zhang W. et al., 2021). Therefore, we took raw or licorice-processed small-flowered and medium-flowered specifications of EF to make extract solutions, intervened in mice at a 5-fold clinically equivalent dose, and examined liver inflammation and oxidative stress indicators and intestinal mucosal microbiota. Our results will contribute to the elucidation of the toxicological properties and the detoxification mechanisms of licorice processing on different specifications of EF.

## Materials and methods

### Drugs and reagents

Processing of licorice: Mix licorice juice with raw EF in a ratio of 3:50, stir well, and stir-fry over low heat until dry (Zhang et al., 2018). EF dry extract: the raw small-flowered (fruit diameter less than 3 mm) and medium-flowered EF (fruit diameter 3 to 5mm), and their licorice-processed were crushed to powder and sieved, weighed equal amounts of powder, respectively, with 12 times the amount of water, refluxed and extracted three times, each time 40 min, combined two filtrates, 50°C water bath concentrated to powder, set aside. Table 1 shows the yield and the amount of raw drug of the four dried extracts of EF, along with the content of limonin and evodiamine in each. The general feed was provided by the laboratory animal center of the Hunan University of Chinese Medicine and produced by Jiangsu Medison Biomedical Co. Ltd. Malondialdehyde (MDA), superoxide dismutase (SOD), tumor necrosis factor-α (TNF-α), interleukin-1β (IL-1β), and interleukin-6 (IL-6) kits were purchased from Konodi Biotechnology Co., Ltd.

**TABLE 1:** Extraction yield of EF dry extract and limonin and evodiamine contents in dry extracts.

Sample	The amount of raw herbs per g of dry extract(g)	Limonin content in dry extract (mg/g)	Evodiamine content in dry extract (mg/g)
Raw small-flowered EF	3.5721	15.76	0.67
Raw medium-flowered EF	2.4040	18.97	0.78
Licorice-processed small-flowered EF	2.0613	8.84	0.43
Licorice-processed medium-flowered EF	2.7686	15.46	0.91

### EF extract solution configuration

Referring to the literature (Zhang et al., 2018), the daily dose for mice was calculated using 5 times the pharmacopoeial dosage (25 g/d) of dried EF extract converted into raw drug quantity and body surface area of mice. Different specifications of EF extract powder were weighed and mixed with a certain amount of distilled water to obtain solutions of EF extract with varying specifications, which were prepared and used immediately. The raw small-flowered EF extract solution (0.91 g/kg⋅d), raw medium-flowered EF extract solution (1.35 g/kg⋅d), licorice-processed small-flowered EF (1.58 g/kg⋅d), licorice-processed small-flowered EF extract solution (1.17 g/kg⋅d).

### Animals

Twenty-five SPF-grade male (Zhou et al., 2024) Kunming mice weighing 20 ± 2 g were provided by Hunan Slaccas Jingda Laboratory Animal Co., Ltd., with license number SCXK2019-0004. All animal procedures were performed in accordance with the Guidelines for Care and Use of Laboratory Animals of Hunan University of Chinese Medicine and approved by the Animal Experimental Ethics Committee of Hunan University of Chinese Medicine (Ethics No: LL2022112302).

### Animal grouping and administration

**FIGURE 1:**
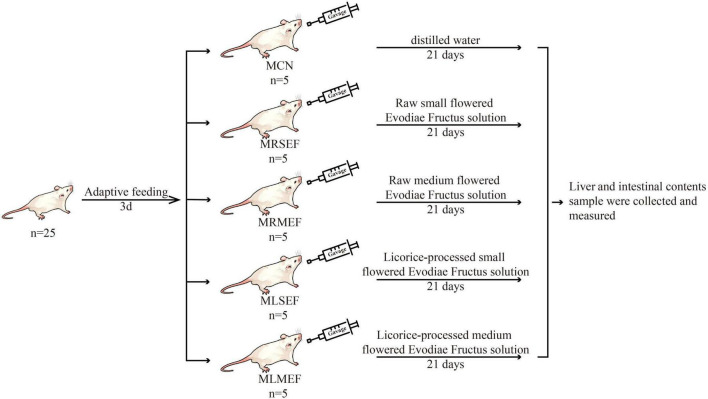
Animal experiment process. MCN: Control group; MRSEF: Raw small-flowered EF group; MRMEF: Raw medium-flowered EF group; MLSEF: Licorice-processed small-flowered EF group; MLMEF: Licorice-processed medium-flowered EF group.

After 3 days of adaptive feeding, 25 mice were randomly divided into the following groups: control (MCN), raw small-flowered EF (MRSEF), raw medium-flowered EF (MRMEF), licorice-processed small-flowered EF (MLSEF), and licorice-processed medium-flowered EF (MLMEF) group. The experimental procedure is shown in Figure 1. The MRSEF, MRMEF, MLSEF, and MLMEF groups were gavaged with extract solutions of raw small-flowered EF, raw medium-flowered EF, licorice-processed small-flowered EF, and licorice-processed medium-flowered EF, respectively. The administration was performed twice a day for 21 consecutive days, 0.35 ml/ time. The MCN group was gavaged with distilled water at the same frequency and amount.

### Biochemical index detection

After 21 days of intervention with EF extract solution, euthanasia was performed on all mice through rapid cervical dislocation. The liver tissues weighing 0.1 g were collected and placed in 1.5 ml centrifuge tubes, followed by the addition of 0.9 ml of physiological saline. The mixture was homogenized at 4°C. After centrifugation at 1000 g for 20 min, the supernatant was collected. According to the instructions provided with the assay kit, the levels of MDA, SOD, TNF-α, IL-1β, and IL-6 in the liver tissues were measured (Wu Y. et al., 2022).

### Intestinal mucosa collection

The mice were dissected under aseptic conditions, the jejunum to ileum section of the intestine was removed and cut with surgical scissors, rinsed in saline to remove the attached contents, placed on sterile filter paper to remove water, and the intestinal mucosa was scraped on the weighing paper with a slide. The scraped intestinal mucosa was put into a sterile tube, weighed, and stored at −80°C (Li et al., 2023).

### DNA extraction and PCR amplification

Total microbial genomic DNA was extracted from each sample tube according to the OMEGA Soil DNA Kit instructions. The quality and concentration of DNA were assessed using 1.0% agarose gel electrophoresis and a NanoDrop^®^ ND-2000 spectrophotometer (Thermo Scientific Inc., USA), respectively (Zhou et al., 2022).

The PCR amplification region is the V3-V4 region of the 16S rRNA gene, with the forward primer 338F (5′-ACTCCTACGGGAGGCAGCA-3′) and the reverse primer 806R (5′-GGACTACHVGGGTWTCTAAT-3′). The samples were purified using the OMEGA DNA purification column, and checked by 1.8% agarose gel electrophoresis. The PCR products were recovered using the Monarch DNA Recovery Kit. The amplification reaction system and conditions refer to our previously published literature (Liu et al., 2023). Finally, the PCR products were sequenced using the Illumina Novaseq 6000 sequencing platform. Shanghai Personal Biotechnology Co. performed the sequencing work.

### Bioinformatics analysis

The raw sequencing data was processed through modifications, trimming, filtering, denoising, merging, and removal of chimeras by the DADA2 method to obtain high-quality sequences (Callahan et al., 2016). The obtained data was analyzed and visualized using QIIME2 (v2019.4) (Caporaso et al., 2010) and R language (v4.2.0). Alpha diversity of the microbial community was assessed using Chao1, Observed species, Shannon, and Simpson indices. Beta diversity of the microbial community was evaluated using non-metric multidimensional scaling (NMDS) analysis. Random forest analysis was employed to rank the importance of the microbial community and identify the key core species in each group. Subsequently, the correlation analysis based on the Spearman correlation coefficient was performed to investigate the relationship between the biochemical indicators in the liver and the core genus. Finally, functional analysis of the gut microbiota in mice intestinal mucosa was conducted using the PICRUSt2 software (v2.2.0-b) (Langille et al., 2013), which provided predicted metabolic function information of different bacterial samples.

### Statistical analysis

The data were statistically analyzed using SPSS 22.0 software. When the measurement data conformed to normal distribution and met variance homogeneity, one-way ANOVA was used to compare the differences between multiple groups, and the Least Significant Difference (LSD) method was selected for two-way comparison between groups. When the data did not conform to normal distribution or the variance was not uniform, the Kruskal Wallis test with Bonferroni correction was used in the nonparametric test, and *P* < 0. 05 indicated that the differences were statistically significant (Yi et al., 2023).

## Results

### Effect of licorice-processed EF extract solution on liver oxidative stress and inflammatory factors

**FIGURE 2:**
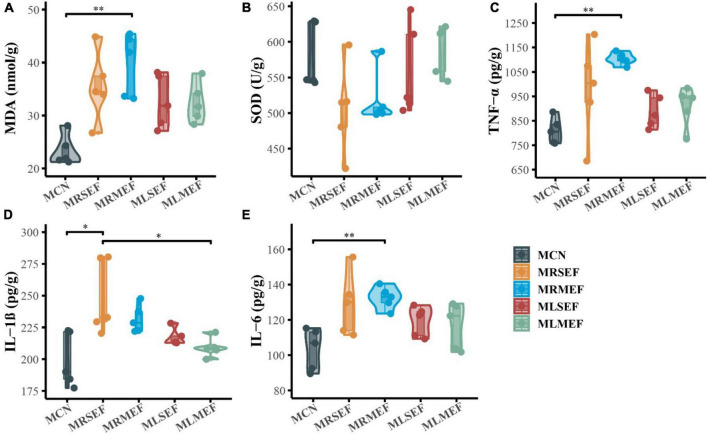
Liver oxidative stress and inflammatory factor levels. **(A)** MDA content. **(B)** SOD content. **(C)** TNF-α content. **(D)** IL-1β content. **(E)** IL-6 content. MCN: Control group; MRSEF: Raw small-flowered EF group; MRMEF: Raw medium-flowered EF group; MLSEF: Licorice-processed small-flowered EF group; MLMEF: Licorice-processed medium-flowered EF group. *P < 0.05, **P < 0.01.

As shown in Figures 2A, B, the MDA content in the liver of mice increased after the intervention of EF extract solution, with the highest content in the MRMEF group, which was significantly higher than the MCN group (*P* < 0.01), indicating that the oxidation products in the MRMEF group were increased. SOD content was opposite to MDA content, and SOD content in the liver of mice decreased after treatment with EF extract solutions, with greater decreases in the MRSEF and MRMEF groups, with no significant difference. Compared with MCN, the inflammatory factors in the MRSEF, MRMEF, MLSEF and MLMEF groups all showed increasing trends (Figures 2C–E). TNF-α and IL-6 contents were significantly higher in the MRMEF group than that in the MCN group (*P* < 0.01), and IL-1β contents were significantly higher in the MRSEF group than in the MCN and MLMEF groups (*P* < 0.01). In contrast, there was no significant difference between the two groups MLSEF, MLMEF and the MCN group (*P* > 0.05). This indicates that the intervention of raw EF increased the level of oxidative stress and promoted the level of inflammatory factors in the liver of mice. Among them, medium-flower EF caused the most damage to the liver.

### Analysis of sequencing quality and number of amplicon sequence variants (ASVs)

**FIGURE 3:**
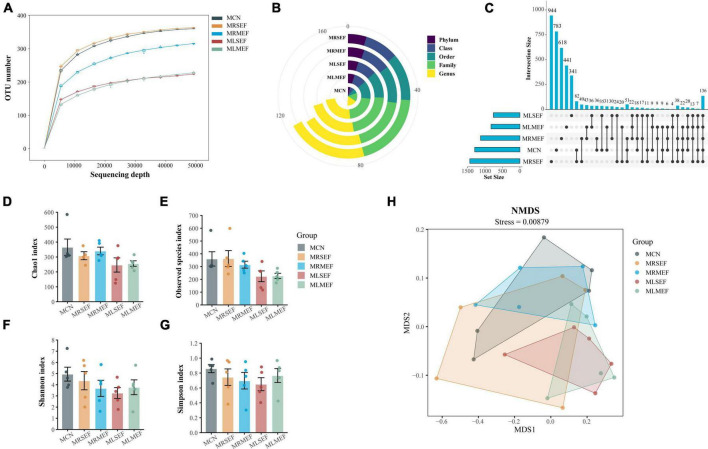
Analysis of ASV number and diversity of intestinal mucosal microbiota. **(A)** Dilution curve of Observed species. **(B)** Number of classifications for each level. **(C)** Up-set plot. **(D)** Chao1 index. **(E)** Observed species index. **(F)** Shannon index. **(G)** Simpson index. **(H)** NMDS analysis. MCN: Control group; MRSEF: Raw small-flowered EF group; MRMEF: Raw medium-flowered EF group; MLSEF: Licorice-processed small-flowered EF group; MLMEF: Licorice-processed medium-flowered EF group.

Based on the dilution curve (Figure 3A), it is evident that with increasing sequencing depth, the number of detected species in each sample has reached a plateau, indicating that the current samples are adequate for subsequent analysis. Moreover, at various taxonomic levels (Figure 3B), the overall classification count in the control group is consistently lower than that in the four EF extract intervention groups. Using Qiime software, sequences were clustered into ASVs based on a 100% sequence similarity threshold. The Up-Set plot illustrates the shared or unique ASV counts among the groups. As depicted in Figure 3C, the groups’ ASV counts are ranked as follows: MRSEF (1436) > MCN (1294) > MRMEF (1128) > MLMEF (828) > MLSEF (761). These findings preliminarily suggest that licorice-processed EF further diminishes species richness in the mouse intestinal mucosa.

### Effect of licorice-processed EF extract solution on the diversity of intestinal mucosal microbiota

We evaluated the species richness of different groups using the Chao1 and Observed species indices, and assessed species diversity using the Shannon and Simpson indices. As shown in Figures 3D–G, except for the MRSEF group, the MRMEF, MLSEF, and MLMEF groups exhibited varying degrees of decline in the Chao1, Observed species, Shannon, and Simpson indices compared to the MCN group. Among them, the MLSEF and MLMEF groups showed the largest decrease in intestinal mucosal richness indices, and the MLSEF group had the lowest values for all four diversity indices among the five groups. These results indicate that EF extract reduced species richness and diversity in the mouse intestinal mucosa. Furthermore, licorice processing exacerbated the decrease in intestinal mucosal microbial richness, with a greater impact observed in small-flower EF.

Subsequently, we used NMDS analysis to evaluate the distribution characteristics of different microbial communities. In NMDS analysis, a smaller Stress value is better, and values below 0.2 are generally considered acceptable (Wu Z. et al., 2022). As shown in Figure 3H, the Stress value for this analysis was 0.00879. Compared to the other groups, the distribution areas of MCN overlapped with the MRSEF and MRMEF groups, while the distribution areas of MCN were relatively separated from the MLMEF and MLSEF groups. This indicates that the EF extract solution intervention did not alter the microbial community structure in the intestinal mucosa. However, licorice-processed EF exhibited a certain degree of regulatory effect on the mouse intestinal mucosal microbiota.

### Effects of licorice-processed EF extract solution on the taxonomic composition and core genera of the intestinal mucosal microbiota

**FIGURE 4:**
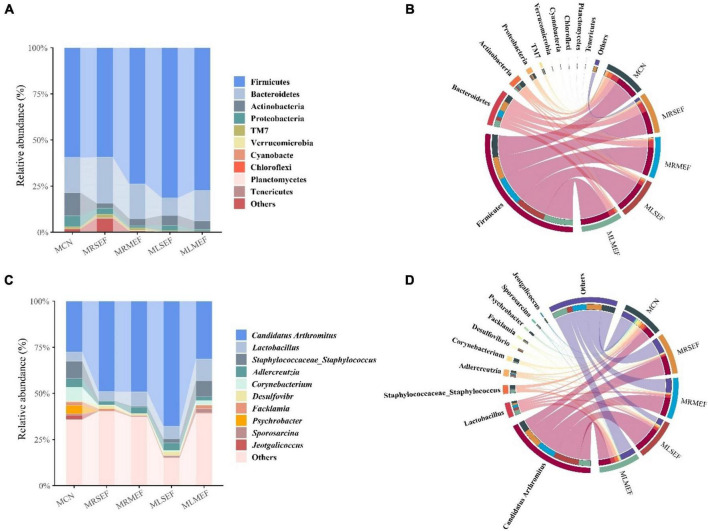
Taxonomic composition of intestinal mucosal microbiota. **(A)** Relative abundance at the phylum level. **(B)** Chord diagram of the phylum level. **(C)** Relative abundance at the genus level. **(D)** Chord diagram of the genus level.

By ASVs analysis and species annotation, a total of 23 distinct phyla and 326 genera were identified. We selected the top 10 abundant phyla and genera for graphical representations (Figures 4A–D). As shown in Figures 4 A, B, Firmicutes (70.24%) and Bacteroidetes (17.83%) dominated the majority of the samples. Compared to MCN (78.58%), the abundance of Firmicutes and Bacteroidetes increased in the MRSEF (84.39%), MRMEF (92.65%), MLSEF (90.96%), and MLMEF (93.87%) groups. Following intervention with EF extract solution, the dominance of the dominant phyla in the intestinal mucosal microbiota was further expanded. At the genus level (Figures 4C, D), *Candidatus Arthromitus* was the most abundant genus in the intestinal mucosa. Compared with MCN (27.59%), the relative abundance of *Candidatus Arthromitus* increased to different degrees in the MRSEF (48.97%), MRMEF (49.17%), MLSEF (67.83%), and MLMEF (31.38%) groups.

**FIGURE 5:**
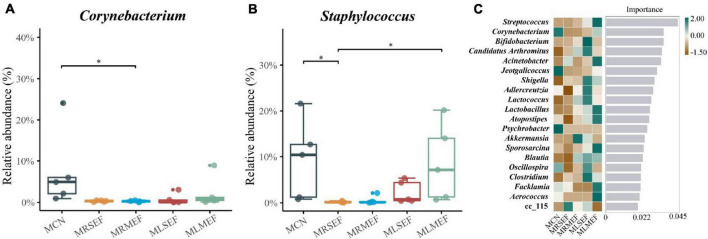
The effect of licorice-processed EF extract solution on the core genera of the intestinal mucosal microbiota. **(A)** Differences in the relative abundance of *Corynebacterium*. **(B)** Differences in the relative abundance of *Staphylococcus*. **(C)** Random forest analysis. The heat map of the relative abundance distribution of the genera in each group is shown on the left. MCN: Control group; MRSEF: Raw small-flowered EF group; MRMEF: Raw medium-flowered EF group; MLSEF: Licorice-processed small-flowered EF group; MLMEF: Licorice-processed medium-flowered EF group. *P < 0.05.

In addition, the relative abundance of *Corynebacterium* and *Staphylococcus* decreased in all four groups (Figures 5A, B). Specifically, the relative abundance of *Corynebacterium* in the MRMEF was significantly lower than that in the MCN group (*P* < 0.05), and the relative abundance of *Staphylococcus* in the MRSEF group was significantly lower than that in the MCN group (*P* < 0.05). Although both *Staphylococcus* and *Corynebacterium* also showed a decreasing trend in the MLSEF and MLMEF groups, there was no significant difference in their relative abundance compared to the MCN group (*P* > 0.05). Licorice processing can alleviate the changes of these two genera of bacteria in the intestinal mucosa induced by the administration of EF to a certain extent.

We further screened the top-ranking core bacterial genera in each group by constructing a random forest model. As illustrated in Figures 5C, the core genera in the MCN group included *Corynebacterium*, *Jeotgalicoccus*, and *Psychrobacter*. A genus under the Erysipelotrichaceae, cc_115, contributed more to the MRSEF group, and *Akkermansia* contributed more to the MRMEF group. The core genera of the MLSEF group include *Bifidobacterium*, *Candidatus Arthromitus*, *Shigella*, *Adlercreutzia*, *Lactococcus*, *Clostridium*, *Oscillospira*, and *Blautia*. The core genera of the MLMEF group include *Streptococcus*, *Acinetobacter*, *Lactobacillus*, *Atopostipes*, *Sporosarcina*, *Facklamia*, and *Aerococcus*.

### Effect of licorice-processed EF extract solution on intestinal microbiota functionality

**FIGURE 6:**
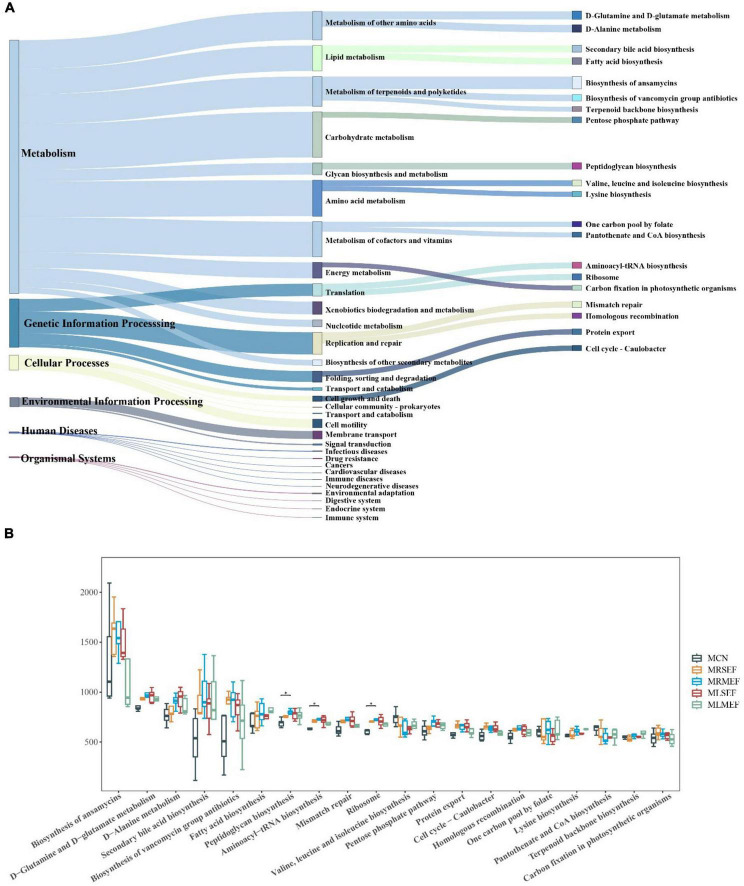
The effect of EF extract solution on intestinal microbiota functionality. **(A)**: Sankey diagram of KEGG function prediction. The first column shows the six classes of primary metabolic pathways, the second column shows the secondary metabolic functions, and the third column shows the tertiary metabolic functions in the top twenty in abundance in mouse intestinal mucosa. **(B)** Difference diagram of metabolic functions in the top 20 in abundance. MCN: Control group; MRSEF: Raw small-flowered EF group; MRMEF: Raw medium-flowered EF group; MLSEF: Licorice-processed small-flowered EF group; MLMEF: Licorice-processed medium-flowered EF group. *P < 0.05.

Based on the intestinal microbiota data, we conducted a functional analysis of the metabolic pathways involved in the intestinal mucosal microbiota using the Kyoto Encyclopedia of Genes and Genomes (KEGG) database. As shown in Figure 6A, metabolism was identified as the predominant primary functional category within the mouse intestinal mucosal microbiota. The primary functional category was further divided into 31 secondary functional categories. Among them, carbohydrate metabolism, amino acid metabolism, metabolism of cofactors and vitamins, metabolism of terpenoids and polyketides, metabolism of other amino acids, and lipid metabolism exhibited the highest abundances. The secondary functional categories were further divided into 182 sub-functions, of which we selected and presented the top 20 abundant functions. These functions may represent the main pathways affected by the administration of EF extract solution within the mouse intestinal mucosal microbiota, including biosynthesis of ansamycins, D-glutamine and D-glutamate metabolism, D-alanine metabolism, secondary bile acid biosynthesis, and biosynthesis of vancomycin group antibiotics.

Based on Figure 6B, compared to the MCN, the MRSEF and MRMEF groups showed increased abundances in 17 out of 20 level 3 functions, with the exceptions of valine, leucine and isoleucine biosynthesis, pantothenate and CoA biosynthesis, and terpenoid backbone biosynthesis. Notably, the MRMEF group exhibited significantly higher abundance in functions such as peptidoglycan biosynthesis, aminoacyl-tRNA biosynthesis, and ribosome compared to the MCN group (*P* < 0.05). After intervention with licorice-processed EF extract solution, the MLSEF and MLMEF showed similar abundance to the MCN group in functions such as biosynthesis of ansamycins, biosynthesis of vancomycin group antibiotics, mismatch repair, ribosome, homologous recombination, and carbon fixation in photosynthetic organisms. Among them, the MLMEF group demonstrated more consistent effects on intestinal mucosal metabolic functions after processing. Compared to the MRMEF, the MLMEF group showed a closer resemblance to the MCN group in the abundance of 14 functions, including biosynthesis of ansamycins, D-glutamine and D-glutamate metabolism, and D-alanine metabolism. This suggests that excessive raw EF extract solution disrupts the metabolic functions of the mouse intestinal mucosa, while processing with licorice can alleviate the impact of raw EF on mouse intestinal mucosal metabolic functions.

### Correlation analysis of intestinal mucosal microbiota with liver biochemical indicators and metabolic functions

**FIGURE 7:**
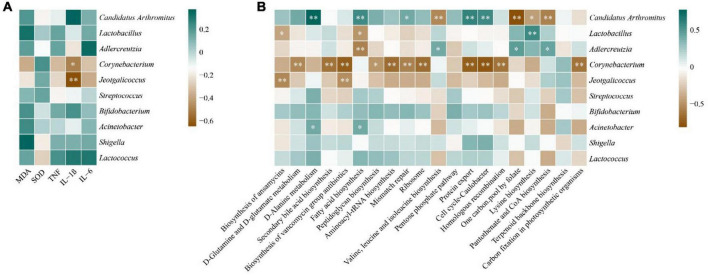
Correlation analysis. **(A)**: Heat map of correlation between core genera and liver biochemical indices. **(B)** Heat map of correlation between core genera and metabolic functions. *P < 0.05, **P < 0.01.

In order to explore the correlation between intestinal mucosal microbiota and liver biochemical indexes and their functions, we did the correlation analysis between the top 10 genera of random forest contribution and liver biochemical indexes and the top 20 abundant intestinal mucosal flora functions in Figure 6. In Figure 7A, MDA and SOD were positively correlated with *Lactobacillus*, *Streptococcus* and *Acinetobacter*, and TNF-α, IL-1β and IL-6 were positively correlated with *Bifidobacterium*, *Shigella* and *Lactococcus*, and with *Corynebacterium* and *Jeotgalicoccus* were negatively correlated. Among them, IL-1β was significantly negatively correlated with *Corynebacterium* and *Jeotgalicoccus* (*P* < 0.05).

In Figure 7B, *Candidatus Arthromitus* showed significant positive correlations with d-alanine metabolism, fatty acid biosynthesis, mismatch repair, protein export, and cell cycle-caulobacter (*P* < 0.05). It showed significant negative correlations with valine, leucine and isoleucine biosynthesis, one carbon pool by folate, lysine biosynthesis, and pantothenate and CoA biosynthesis (*P* < 0.05). *Acinetobacter* exhibited a significant positve correlation with D-alanine metabolism and fatty acid biosynthesis. *Lactobacillus* demonstrated significant negative correlations with the biosynthesis of ansamycins and fatty acid biosynthesis, but a significant positive correlation with lysine biosynthesis (*P* < 0.05). *Adlercreutzia* showed a significant negative correlation with fatty acid biosynthesis, but significant positive correlations with valine, leucine and isoleucine biosynthesis, one carbon pool by folate, and pantothenate and CoA biosynthesis (*P* < 0.05). It is worth noting that *Corynebacterium* displayed significant negative correlations with several important metabolic functions, including D-glutamine and D-glutamate metabolism, secondary bile acid biosynthesis, and biosynthesis of vancomycin group antibiotics (*P* < 0.01).

## Discussion

Oxidative stress is typically caused by an imbalance between reactive oxygen species and the body’s antioxidant defense system, and it is one of the most common mechanisms of cell death in organ injuries, including liver damage (Pizzino et al., 2017). Among them, MDA is the final product of lipid oxidation, while SOD is an antioxidant enzyme and one of the body’s cellular defense mechanisms against oxidative stress. Therefore, the balance between them can reflect the level of oxidative stress response, which affects liver health. After intervention with raw EF extract solution, SOD levels had no difference before and after administration of EF. MDA level significantly increased in MRMEF, meanwhile, there was no difference in MDA level between MC and MLMEF. It means that the intervention of raw medium-flowered EF caused liver damage in mice, and licorice processing mitigated the damage. Licorice-processed EF relieves the damage degree of the liver by reducing lipid peroxidation. It remains to be further studied that whether licorice-processed EF can cause other changes in the molecular mechanisms of oxidative stress.

TNF-α, IL-1β and IL-6 are all pro-inflammatory cytokines, and they play complex roles in the inflammatory response, that can both help the host defend against infection and promote inflammation. Their increased levels contribute to the development of inflammation, thus mediating liver injury. In a recent report, (Zhang M. et al., 2021) used EF of different doses to intervene in rats and found that licorice processing significantly reduced the levels of TNF-α, IL-1β, and IL-6 in serum by EF. We found a similar pattern that the magnitude of changes in inflammation markers decreased after intervention with licorice-processed EF solution, indicating that licorice processing has a certain alleviating effect on the liver inflammatory damage caused by EF intervention. Interestingly, we also found the differences in the inflammatory response mediated by different sizes of EF. In Figures 2C–E, IL-1β significantly increased in MRSEF, while TNF-α, and IL-6 levels significantly increased in MRMEF. This may be related to the difference in chemical composition in different sizes of EF. And licorice processing narrows down the differences in liver damage caused by different specifications.

Despite the intervention of the raw EF and licorice-processed EF, there was no obvious change in the structure of the microbiota (Figures 3D–G). However different sizes of EF before and after processing caused differences in the core bacterial genera, which may penetrate their inflammatory reactions. Following the intervention of EF extract solution, except for the MLMEF group, the abundance of *Candidatus Arthromitus* in the intestinal mucosa increased. Among them, the MLSEF group exhibited the highest abundance. *Candidatus Arthromitus* is one of the most abundant genera in the intestinal mucosal microbiota. Previous studies have indicated that *Candidatus Arthromitus* potentially plays a role in promoting the growth of probiotics and shaping adult intestinal metabolism (Hedblom et al., 2018; Vallianou et al., 2021). Wu et al. (2020) reported that the processing of licorice can reduce the hepatotoxicity of *Tripterygium wilfordii*, and this mechanism may be associated with the modulation of fatty acid metabolism. In the correlation analysis (Figure 6B), *Candidatus Arthromitus* showed significant positive correlations with metabolic pathways including fatty acid biosynthesis, D-alanine metabolism, and mismatch repair. In addition, probiotics such as *Bifidobacterium* (He et al., 2019), *Adlercreutzia* (Ziętak et al., 2016), *Lactococcus* (Casalta and Montel, 2008), *Oscillospira* (Yang et al., 2021), and *Blautia* (Liu et al., 2021) were enriched in the MLSEF group. This indicated that licorice processing small-flower EF could improve the intestinal microbiota by promoting probiotics such as *Candidatus Arthromitus*, *Bifidobacterium*, and thus regulating fatty acid metabolism.

However, this effect may change with the gradual ripening of EF. In terms of medium-flower EF, the maximum enrichment of *Lactobacillus* genus was observed in the MLMEF group. *Lactobacillus* may be the key genus of licorice for regulating intestinal microbiota according to the previous findings of Wang et al. (2020). The majority of *Lactobacillus* is recognized as probiotics, exhibiting functions such as maintaining intestinal mucosal integrity, modulating immunity, and alleviating gastrointestinal inflammation (Heeney et al., 2018; Ji et al., 2020). Research has suggested that the therapeutic effects of Evodiamine on ulcerative colitis associated with an increase in *Lactobacillus acidophilus* abundance and acetate production (Wang M. X. et al., 2021). Therefore, the alleviation of inflammation in processed medium-flowered EF may be attributed to the elevation of *Lactobacillus* abundance. Despite the positive correlations between *Candidatus Arthromitus*, *Bifidobacterium*, and *Lactobacillus* with MDA, IL-1β, TNF-α, and IL-6 levels, opportunistic pathogens such as *Shigella*, *Staphylococcus*, and *Acinetobacter* were also enriched in the MLSEF or MLMEF groups (Qi, 2010; Amorim and Nascimento, 2017). Licorice processing only mitigated the liver inflammation of EF, and the presence of these opportunistic pathogens may be associated with the induction of inflammation under excessive intervention of EF. Moreover, compared with the MCN group, *Corynebacterium* and *Jeotgalicoccus* exhibited lower abundance in all four EF solution intervention groups. *Jeotgalicoccus* is a natural producer of terminal olefins, with limited clinical reports (Nusantara Putra et al., 2019). *Corynebacterium* is an opportunistic pathogen commonly found in immunocompromised patients (Sengupta et al., 2015; Kalt et al., 2018). It is significantly and negatively associated with several dominant metabolic functions, including peptidoglycan biosynthesis, aminoacyl-tRNA biosynthesis, and ribosome, which are significantly elevated in the MRMEF group. Furthermore, *Corynebacterium* is negatively correlated with MDA, TNF-α, and IL-6, and significantly negatively correlated with IL-1β. Compared to the intervention group of raw EF, the abundance of *Corynebacterium* is higher in licorice-processed EF. This aligns with the previous mention that licorice may cause moderate increases in inflammatory factors to exert its anti-infective effects (Wang et al., 2020). This suggests that the addition of licorice juice is the key to influencing the intestinal bacteria and pharmaceutical effect of EF. Licorice-processed EF not only reduces the pathogenic bacteria in the intestinal mucosal microbiota and adjusts the balance of the intestinal mucosal microbiota, but also enhances the mice’s resistance to infection by regulating the abundance of *Corynebacterium*.

As one of the most commonly used herbal medicines in Asia, the reliability of EF therapeutic efficacy has been tested for centuries (Li and Wang, 2020). Licorice-processed EF is commonly used in clinical preparation, widely approved as less toxic than raw products. Its toxicity is suspected to be size related, which is still far from certain. Therefore, investigating the scientific connotation of processing different specifications of EF with licorice to reduce its toxicity is of significant importance for promoting the use of EF and ensuring patient safety.

## Conclusion

Based on the above, excessive intervention with raw EF extract solution resulted in liver oxidative damage and increased levels of inflammatory factors in mice. The effect of medium-flowered EF on oxidative stress and inflammatory factors in the liver of mice was stronger than that of small-flowered EF. Licorice processing can alleviate the liver inflammation caused by different sizes of EF extracts, restore intestinal mucosal function closer to the normal group, and potentially enhance the mice’s resistance to infection. This is closely related to the regulation of *Corynebacterium* abundance. Moreover, the licorice processing influenced the modulatory effect of EF on the intestinal microbiota. The licorice-processed small-flowered EF can promote the growth of beneficial bacteria such as *Candidatus Arthromitus* and *Bifidobacterium*, thereby regulating fatty acid metabolism. While licorice-processed medium-flowered EF promotes the growth of the probiotic *Lactobacillus*.

## Data availability statement

The datasets presented in this study can be found in online repositories. The names of the repository/repositories and accession number(s) can be found here: NCBI-PRJNA989095.

## Ethics statement

The animal study was approved by the Animal Experimental Ethics Committee of Hunan University of Chinese Medicine. The study was conducted in accordance with the local legislation and institutional requirements.

## Author contributions

XL: Project administration, Supervision, Writing−original draft, Writing−review and editing. QT: Formal analysis, Resources, Writing−review and editing. LC: Data curation, Methodology, Writing−review and editing. YZ: Investigation, Resources, Writing−review and editing. YP: Funding acquisition, Project administration, Validation, Writing−review and editing.
